# A lightweight dual-attention network for tomato leaf disease identification

**DOI:** 10.3389/fpls.2024.1420584

**Published:** 2024-08-06

**Authors:** Enxu Zhang, Ning Zhang, Fei Li, Cheng Lv

**Affiliations:** Engineering Research Center of Hydrogen Energy Equipment& Safety Detection, Universities of Shaanxi Province, Xijing University, Xi’an, China

**Keywords:** tomato disease identification, machine vision, deep learning, lightweight models, attention mechanisms

## Abstract

Tomato disease image recognition plays a crucial role in agricultural production. Today, while machine vision methods based on deep learning have achieved some success in disease recognition, they still face several challenges. These include issues such as imbalanced datasets, unclear disease features, small inter-class differences, and large intra-class variations. To address these challenges, this paper proposes a method for classifying and recognizing tomato leaf diseases based on machine vision. First, to enhance the disease feature details in images, a piecewise linear transformation method is used for image enhancement, and oversampling is employed to expand the dataset, compensating for the imbalanced dataset. Next, this paper introduces a convolutional block with a dual attention mechanism called DAC Block, which is used to construct a lightweight model named LDAMNet. The DAC Block innovatively uses Hybrid Channel Attention (HCA) and Coordinate Attention (CSA) to process channel information and spatial information of input images respectively, enhancing the model’s feature extraction capabilities. Additionally, this paper proposes a Robust Cross-Entropy (RCE) loss function that is robust to noisy labels, aimed at reducing the impact of noisy labels on the LDAMNet model during training. Experimental results show that this method achieves an average recognition accuracy of 98.71% on the tomato disease dataset, effectively retaining disease information in images and capturing disease areas. Furthermore, the method also demonstrates strong recognition capabilities on rice crop disease datasets, indicating good generalization performance and the ability to function effectively in disease recognition across different crops. The research findings of this paper provide new ideas and methods for the field of crop disease recognition. However, future research needs to further optimize the model’s structure and computational efficiency, and validate its application effects in more practical scenarios.

## 1 Introduction

Originating from the indigenous regions of South America, the tomato is a crop with a short growth cycle, low environmental requirements, and rich nutritional value, and has been widely cultivated around the world (Mitchell et al., 2007; Bhatkar et al., 2021). In agricultural production, tomato plants are susceptible to a variety of pathogenic bacteria and environmental factors such as fungi, bacteria, and viruses, resulting in the occurrence of white spot disease, early blight, mosaic virus, leaf mold, and other diseases. These diseases are mainly manifested in the leaves and affect their function, thus affecting the yield and quality of tomatoes. Especially under conditions of frequent rainfall or high humidity, tomato plants are more likely to be infected with diseases, resulting in seedling rot and stem and fruit rot (Vos et al., 2014). However, the diversity and complexity of tomato diseases pose great challenges to control. During the occurrence of these diseases, early symptoms usually appear on tomato leaves, showing abnormal characteristics that are different from those of healthy leaves, as detailed in **Supplementary Table S2**. Early and accurate disease identification in agricultural production can effectively reduce the yield loss caused by diseases. However, traditional manual methods of disease identification are inefficient and often require specialized agricultural expertise, hindering widespread and accurate identification of diseases and resulting in wasted labor and medicines (Patil and Thorat, 2016). Therefore, there is an urgent need for a convenient and rapid detection method that can non-destructively identify plant developmental abnormalities at an early stage to mitigate the impact of diseases on agricultural production (Eli-Chukwu and Ogwugwam, 2019). Nowadays, with the rise of precision agriculture and smart agriculture concepts, it is important to use machine vision technology to assist agricultural production, realize the accurate identification of tomato diseases, take management measures and prevention strategies in a timely manner, and improve crop yields (Affonso et al., 2017).

Identifying plant leaf diseases falls under the field of agricultural information technology. The rapid development and advancement of machine vision technology provide new directions for crop disease identification and combined with robotics technology, can achieve more flexible agricultural production (Tang Y, et al., 2023; Ye et al., 2023). Initially, machine learning algorithms were used to extract image features and classify them. (Xie and He, 2016) used a gray-level co-occurrence matrix to extract texture features and classified them using the K-NN algorithm. (Akbarzadeh et al., 2018) employed support vector machines to efficiently distinguish weeds based on the morphological features of broadleaf and narrow plants. However, using traditional machine learning algorithms for disease identification typically relies on single global features such as color, texture, and shape. This often requires researchers to manually design image feature extraction methods based on experience, resulting in a limited ability to identify various types of diseases and insufficient recognition capability to meet the needs of large-scale agricultural disease identification (Khan et al., 2018).

With the development of deep learning, it has shown significant advantages in feature extraction and recognition tasks. Deep learning-based disease image recognition has become an important method in current research. Convolutional neural network (CNN) models, by introducing operations such as local connections and weight sharing, have made significant progress in various crop disease identification tasks and are currently considered one of the best algorithms for pattern recognition tasks (Prathibha et al., 2017). To address the data imbalance problem in cassava disease detection based on CNN models, (Gnanasekaran and Opiyo, 2020) used methods such as class weights, SMOTE, and focal loss functions to enhance the model’s recognition performance on imbalanced datasets. (Wu, 2021) constructed a dual-channel convolutional neural network model by integrating ResNet50 and VGG19 network models, thereby improving the network model’s ability to extract disease features and achieve high-precision recognition of maize diseases. Additionally, to address the challenge of identifying grape diseases in natural environments, (Cai et al., 2023) used an improved MSR algorithm to process images and employed a Siamese network structure to extract image features, achieving model lightweighting. (Sanida et al., 2023) improved recognition capability by combining VGG and Inception modules and using a multi-scale approach to enhance the model. (Uddin et al., 2024) integrated Inception V3 and DenseNet201 with the addition of the attention mechanism VIT to obtain the E2ETCA network model for rice disease identification. (Deng et al., 2023) used a combination of ResNest and Ghost to obtain GR-ARNet, which separately processed the depth feature information and channels of images, achieving efficient identification of banana leaf diseases. In agricultural production, to effectively prevent and control diseases, (Kamal et al., 2019) proposed a MobileNet model improved by deep separable convolution, which outperformed VGG and GoogleNet models on the 55-class PlantVillage leaf dataset. (Waheed et al., 2020) proposed an optimized DenseNet model for identifying maize leaf diseases.

In neural network models, the attention mechanism is an effective method to improve the model’s recognition performance. Neural network models can use the attention mechanism to compute the weights of input images, selectively emphasizing areas of interest through feature weighting, thereby aiding feature extraction. Currently, many achievements have been made, such as SE-Net (Hu et al., 2018), ECA-Net (Wang et al., 2020), CBAM (Woo et al., 2018), and Coordinate Attention (Hou et al., 2021). Additionally, through the efforts of many researchers, the attention mechanism can be applied to plant disease detection. For example, (Zhao et al., 2022) embedded the CBAM attention mechanism into the Inception network model, thereby enhancing the network model’s ability to identify diseases in maize, potatoes, and tomatoes. (Zeng and Li, 2020) proposed a self-attention convolutional neural network (SACNN) and added it to the neural network, achieving good recognition results on the MK-D2 agricultural disease dataset. (Chen et al., 2021) proposed an attention module (LSAM) for MobileNet V2, effectively enhancing the network’s recognition capability for diseases. (Liao et al., 2023) optimized the network by combining ResNet-50, long short-term memory (LSTM) network, and SE-Net attention mechanism. (Tang L. et al., 2023) proposed a ternary parallel attention module based on the CBAM attention mechanism, combined with a multi-scale hybrid model composed of Inception modules and ResNext modules, achieving good results in the identification of apple leaf diseases. Additionally, some scholars have integrated deep learning network models with robotics technology. For instance, (Wang et al., 2023) integrated a visual system and a robotic intelligent control system, enabling positioning for lychee harvesting and obstacle recognition and avoidance.

In tomato disease identification, there are also numerous research achievements. (Mokhtar et al., 2015) used Gabor wavelet transform to extract image features and then used support vector machines to identify tomato leaf diseases. (Anandhakrishnan and Jaisakthi, 2022) improved the LeNet5 network model architecture, combining support vector machines (SVM) and multilayer perceptron to detect tomato diseases. (Ullah et al., 2023) used a hybrid network approach, combining EfficientNetB3 and MobileNet into the EffiMob-Net multi-scale model to detect tomato leaf diseases. (Zhou et al., 2021) introduced dense network connections into the residual network model, forming a hybrid network model that improved recognition accuracy while achieving model lightweighting. (Zaki et al., 2020) used the PCBAM attention mechanism and Dense Inception convolution blocks to optimize the MobileNet model. (Zhang et al., 2023) proposed a multi-channel automatic direction recursive attention network (M-AORANet) to address noise issues in tomato disease images, effectively achieving disease recognition, although some difficulties remained with other crop diseases. (Chen et al., 2020) combined the ResNet-50 network model with the proposed dual-channel residual attention network model (B-ARNet) to enhance the model’s recognition of tomato diseases from multiple scales. (Zhao et al., 2021) optimized the ResNet50 model by using the SE-Net attention mechanism and combining it with a multi-scale feature extraction module to recognize tomato diseases.

Neural network models are effective for agricultural plant disease identification, and many new and original network structures have emerged in recent years. These network model structures can improve the recognition effect of the model by combining image enhancement algorithms, attention mechanisms and fusion methods. However, due to the uneven spatial distribution of the characteristics of agricultural diseases and the influence of different stages of their onset, the problems of small disease characteristics, large differences in similar characteristics, and small differences in heterogeneous characteristics lead to the difficulty of achieving high precision and lightweight of the model. Therefore, the purpose of this paper is to propose a high-precision detection method with limited computing resources, which can meet the accuracy requirements and be suitable for mobile deployment. The specific contributions of this article are as follows:

Image enhancement technology was used to enhance the detailed features of leaf disease images. Initially, the piecewise linear transformation method was used to remap the brightness values of the image by setting thresholds for minimum and maximum brightness, enhancing the detailed features, and helping the neural network model extract abstract features during training.

A lightweight CNN neural network model, LDAMNet, is proposed, which is mainly composed of a double attention convolution (DAC) block with mixed-channel attention (HCA) and coordinate space attention (CSA) functions. Set the number of blocks by pressing [1,1,3,1] in four different stages.

Considering the influence of noise labels in CNN model training, the Cross-Entropy loss function is improved, and the Robust Cross-Entropy Loss (RCE) loss function is derived by introducing a weighted formula with two adjustable parameters, α and β , which enhances the ability of the model to deal with label noise. In addition, by adjusting these two parameters, the loss value in model training can be flexibly adjusted.

Finally, by comparing different CNN models, different blocks, different normalization methods, as well as ablation experiments and experiments using different datasets, the effectiveness of the proposed method for identifying tomato leaf disease is proved with limited computing resources, and the recognition ability is on par with that of large-scale models, which has obvious advantages compared with existing lightweight models.

The structure of this paper is summarized as follows: the second part mainly introduces the methods used in this paper, including LDAMNet, DAC block, HCA channel attention mechanism, CSA spatial attention mechanism, and RCE loss function. Subsequently, the third part is mainly used to test the detection method proposed in this paper and evaluate the performance of the proposed identification method in all aspects through five different experiments. Finally, the fourth part mainly summarizes the work and experimental conclusions of this paper.

## 2 Materials and methods

### 2.1 Image preprocessing

#### 2.1.1 Sample

The tomato image dataset used in this study is derived from the Plant Disease Classification Merged Dataset published on the Kaggle platform (https://www.kaggle.com/datasets/alinedobrovsky/plant-disease-classification-merged-dataset).

**Figure 1:**
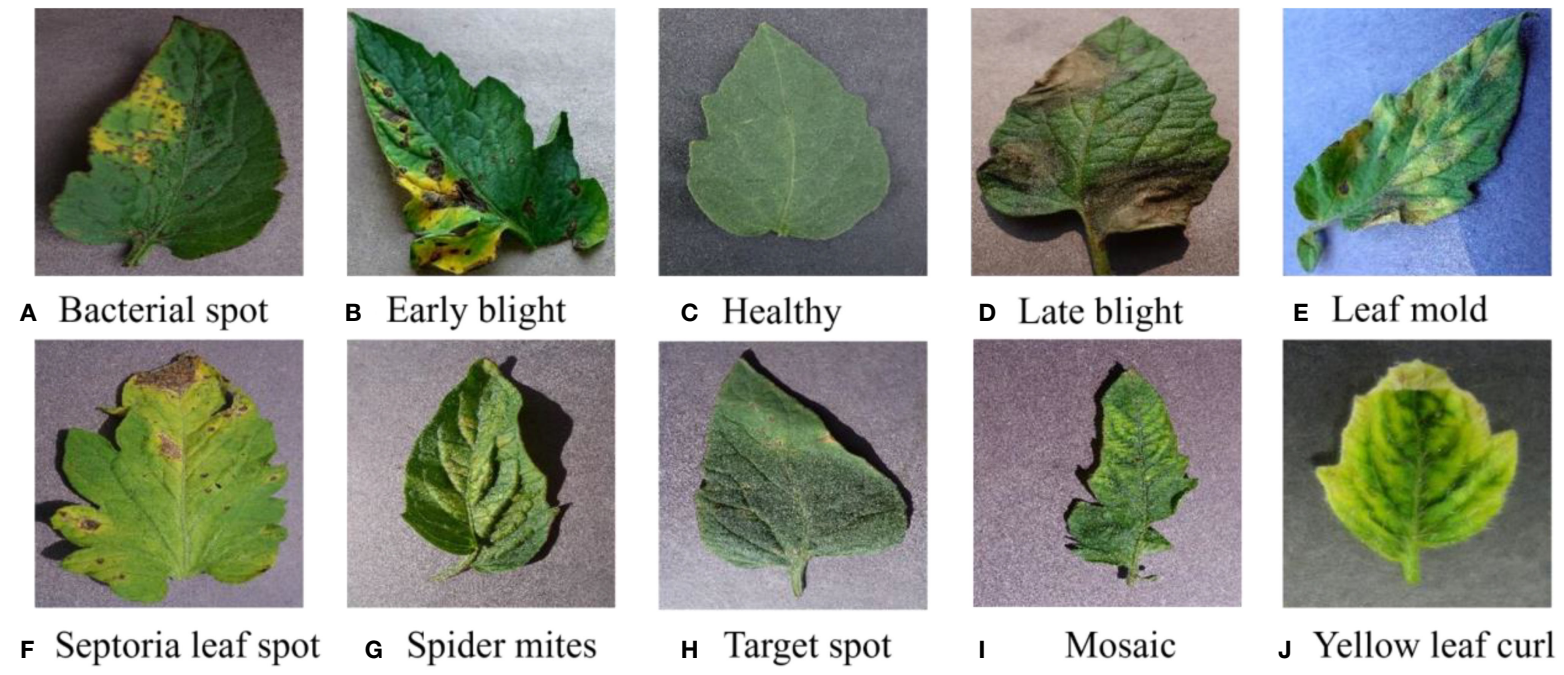
Different categories of leaf images in the tomato dataset. From **(A–J)**, it includes 10 types of disease and healthy leaf image samples.

The dataset combines 14 existing agricultural imagery datasets covering 88 disease categories affecting 23 different crops. In this paper, the tomato leaf disease images were selected as the dataset for the study, including ten disease images at different disease stages. The image size in the dataset is 256×256, the leaf samples are shown in **Figure 1**, and the disease characteristics are shown in **Supplementary Table S2**.

#### 2.1.2 Image processing

Training data plays a vital role in the performance of CNN models, which directly affects the training effect of model training. The process of image acquisition is usually affected by the image acquisition equipment and environment, resulting in problems such as inconsistent brightness and noise. These issues can obscure image features, hinder the model’s ability to discriminate features during training, and ultimately impair its ability to recognize. In addition, due to the different number of disease samples, there are large differences in the number of images of different categories in the image dataset. This will cause the model to tend to the category with a large number of images during the training process, and the images of other categories cannot be effectively recognized, resulting in overfitting (Kong et al., 2021).

**Figure 2:**
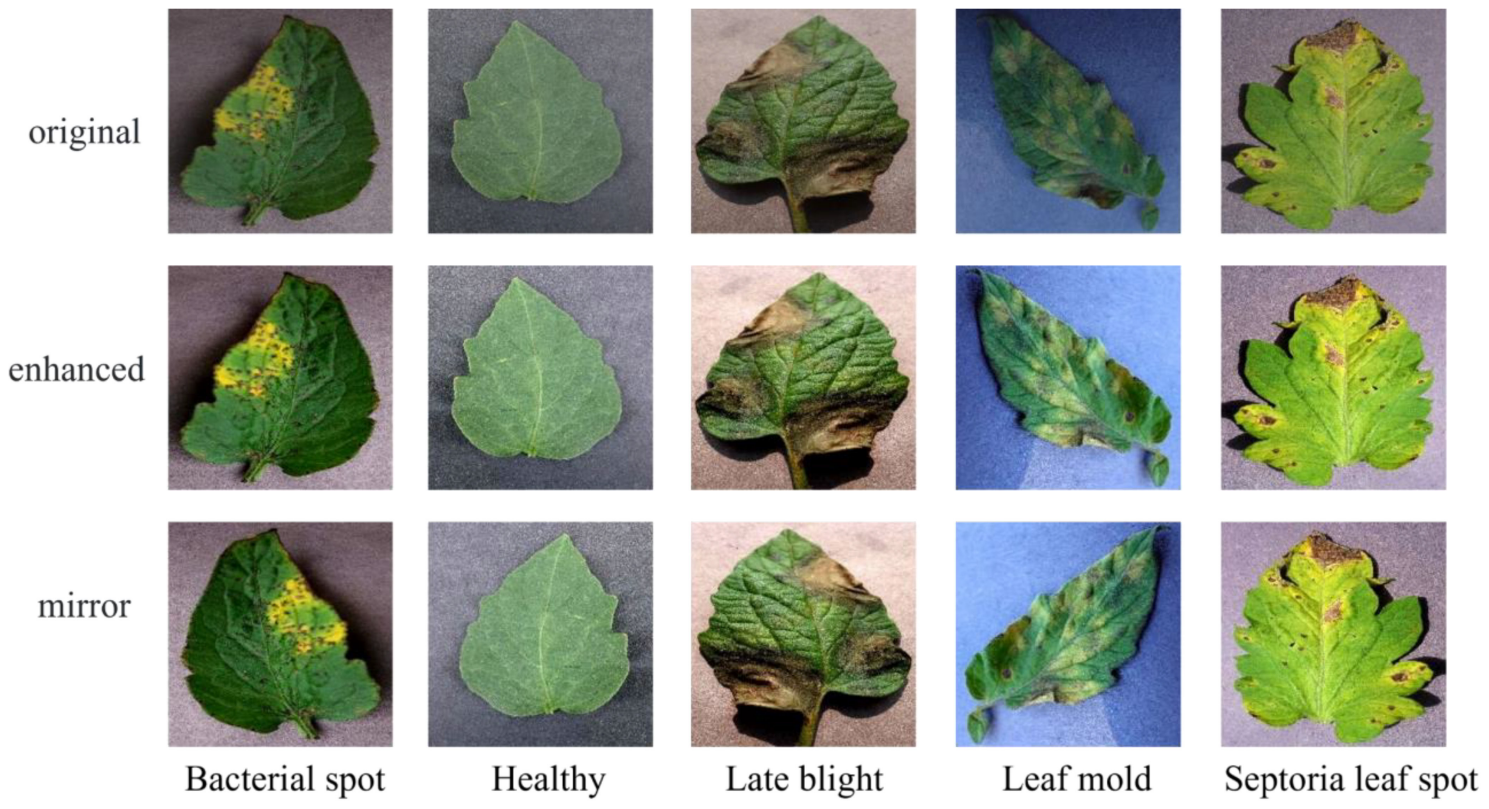
Image-enhanced sample display. Original represents the images in the original dataset, and enchanced and mirror represent enhanced and oversampled image samples, respectively.

To address the aforementioned issues, this paper proposes an image processing method. In this method, images from the dataset are first decomposed into red, green, and blue color channels. Then, based on the set thresholds a and b , as well as the range 0-255, the pixels in each channel are divided into three intervals. The pixel values in different intervals are processed according to Equation 1 to obtain the processed pixel value F(x) . Finally, the three processed color channels are recombined to obtain enhanced disease image samples. Moreover, data imbalance is a crucial factor affecting the training effectiveness of deep learning network models. Network models tend to overly learn features from categories with more samples and struggle to effectively classify categories with fewer samples. To address this, this paper uses oversampling to balance the samples in the image dataset. The effects of image enhancement and oversampling are shown in **Figure 2** The enhanced image dataset is divided into training and test sets at an 8:2 ratio, with 15975 images for training and 3994 for testing. **Table 1** shows the sample distribution before and after data augmentation.

**Table 1:** Tomato leaf disease dataset sample size.

Categories	Before	After	train	test
Bacterial spot	1612	1969	1575	394
Early blight	1000	2000	1600	400
Healthy	1251	2000	1600	400
Late blight	1209	2000	1600	400
Leaf mold	952	2000	1600	400
Septoria leaf spot	1379	2000	1600	400
Spider mites	1257	2000	1600	400
Target spot	988	2000	1600	400
Mosaic	373	2000	1600	400
Yellow leaf curl	1849	2000	1600	400


(1) $$F(x)=\{\begin{array}{c} \begin{array}{cc} 0 & \;x<a \end{array}\\ \begin{array}{cc} \frac{255}{\left(b-a)(x-a\right)} & \;a<x<b \end{array}\\ \begin{array}{cc} 255 & \;x>b \end{array} \end{array}$$

### 2.2 LDAMNet model

**Figure 3:**
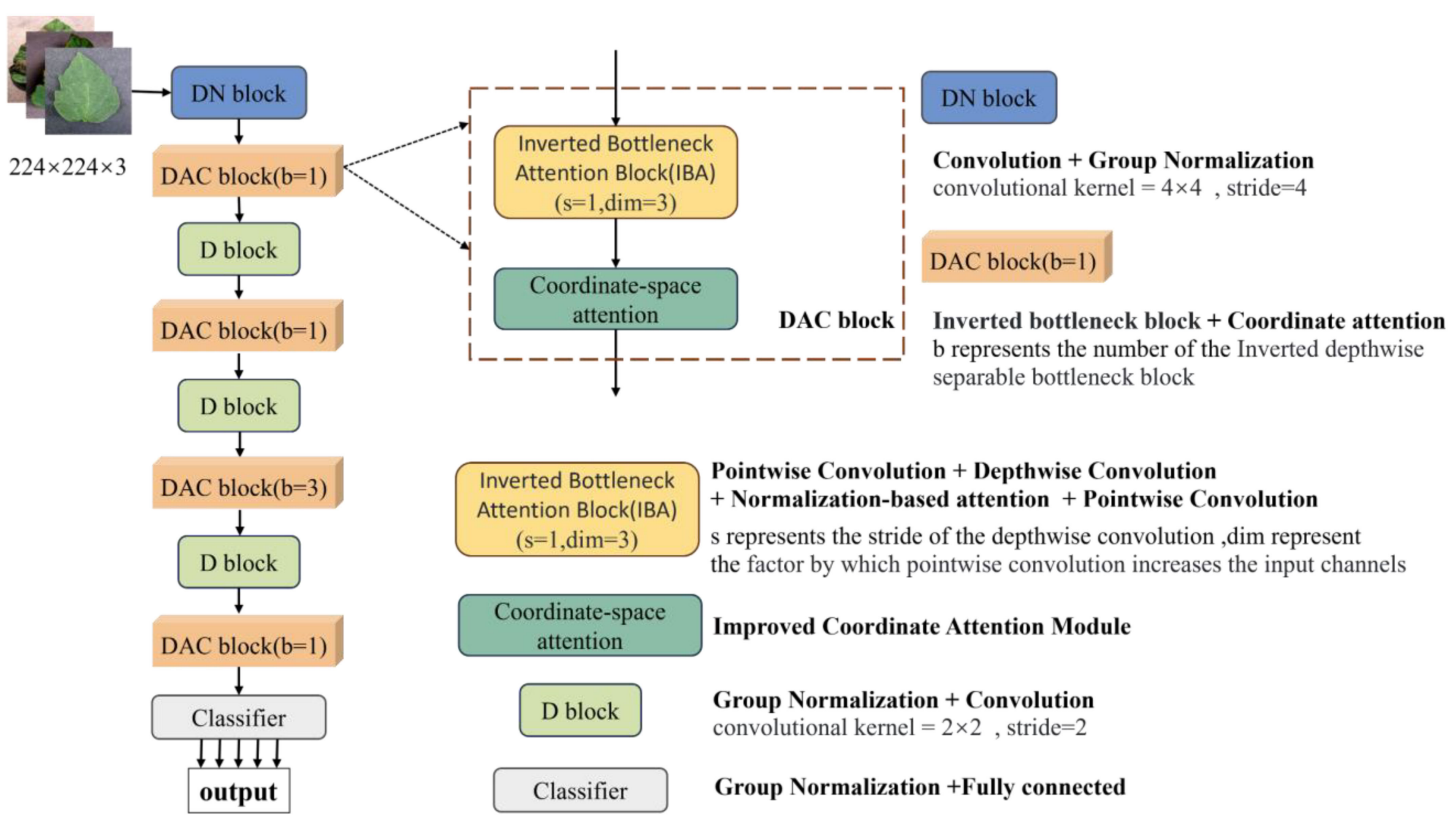
The structure of the LDAMNet model. b, s, and dim represent the number of IBA blocks, the stride of deep convolution, and the multiple of IBA block expansion image channels, respectively.

The LDAMNet model proposed in this paper is used for tomato leaf disease identification and is composed of DN block, DAC block, D block, and Classifier. In model training, the DN block and D block are used to downsample the input image, the DAC block extracts the features of the input image, and finally, the Classifier implements the classification of the image. The DN block is composed of a convolutional layer with a convolutional kernel of 4×4 and GN, which mainly processes the input image to reduce the size of the image and extracts features in a large range by using the convolutional kernel of 4×4. The D block is composed of a convolutional layer with GN and a convolutional kernel of 2×2, which is smaller than the DN block, which makes the network model pay more attention to the local features of the image. The DAC module allocates the number of blocks in four stages according to the quantities 1, 1, 3, 1, and its structure is shown in **Figure 3** and **Table 2**.

**Table 2:** Architectures for LDAMNet.

output size	layer name	LDAMNet
56×56	DN block	Conv(4×4, 32) GroupNorm(channel = 32)
	DAC block	IBA block × 1 CSA block × 1
28×28	D block	GroupNorm(channel = 32) Conv (2×2, 64)
	DAC block	IBA block × 1 CSA block × 1
14×14	D block	GroupNorm(channel = 64) Conv (2×2, 128)
	DAC block	IBA block × 3 CSA block × 1
7×7	D block	GroupNorm(channel = 128) Conv (2×2, 256)
	DAC block	IBA block × 1 CSA block × 1
1×1	Classifier	GroupNorm(channel = 256) Linear(256, 10)

**Figure 4:**
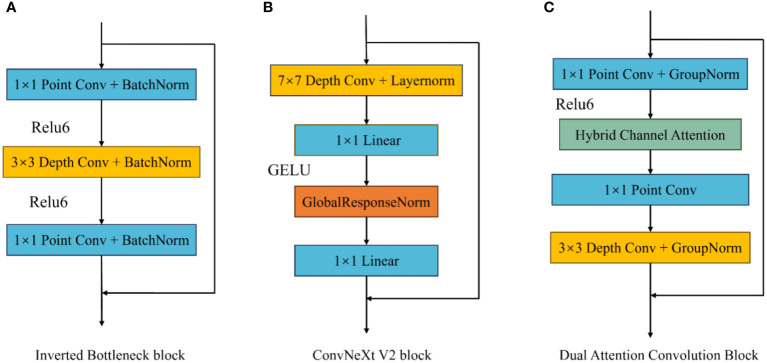
Different inverted bottleneck block structures. **(A)** is the convolution block in MobileNetV2, **(B)** is the convolution block in ConvNextV2, and **(C)** is the IBA Block.

**Figure 5:**
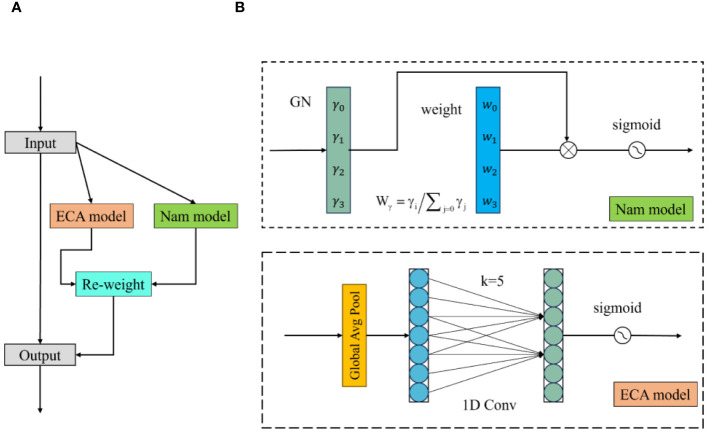
Hybrid channel attention structure. **(A)** is the HCA structure diagram and **(B)** is the NAM and ECAmodel structure diagram.

The IBA block is shown in **Figure 4B**, CSA in **Figure 5**. The DN block and D block have strides of 4 and 2, respectively.

### 2.3 Dual attention convolution block

To enhance the extraction of complex image features, this paper improves the existing inverted bottleneck block and proposes a Dual Attention Convolution (DAC) block. It consists of an Inverted Bottleneck Attention (IBA) block and Coordinate Space Attention (CSA), as shown in **Figure 3** The IBA block mainly comprises two pointwise convolution layers, a 3×3 depthwise convolution layer, Hybrid Channel Attention (HCA), ReLU6, and two GroupNorm layers. CSA is a lightweight coordinate space attention mechanism proposed in this paper to locate regions of interest in images. As shown in **Figure 4C**, the IBA module draws inspiration from the inverted bottleneck module, ConvNeXt V2 module, and Channel Attention module (CBAM), focusing primarily on processing input image channels to enrich disease feature representation.

#### 2.3.1 Inverted bottleneck attention block

The inverted bottleneck block, first applied in MobileNet V2, serves as an optimization method for traditional convolution layers, effectively reducing the computational and parameter requirements for model training. In the inverted bottleneck block, the number of image channels and image size remain unchanged, allowing for effective information extraction while reducing model size. The ConvNeXt V2 block improves upon the inverted bottleneck block by adding a 7×7 convolution layer before the first pointwise convolution to capture broader spatial features and mitigate the impact of complex backgrounds on model recognition performance. Additionally, it uses Global Response Normalization (GRN) instead of depthwise convolution between the two pointwise convolution layers.

This paper improves the inverted bottleneck block by placing the depthwise convolution after two pointwise convolution layers, adding an HCA module and ReLU6 activation function to enhance the model’s expressiveness, and introducing GroupNorm to replace BatchNorm, reducing the model’s dependence on training batch sizes. As shown in **Figure 4C**, after the input image passes through the first pointwise convolution and ReLU6 activation function, the input image channels are expanded according to the size of the parameter ‘dim’ and undergo nonlinear transformation. Then, the HCA channel attention mechanism obtains weights for the expanded channels and applies weighting. After weighing, the channels pass through a second pointwise convolution, preserving channels with rich feature expressions. Finally, a 3×3 depthwise convolution organizes the spatial information of the retained channels, facilitating subsequent CSA extraction of regions of interest in the image.

#### 2.3.2 Hybrid channel attention

EfficientNet and ConvNeXt V2 use SE-Net and Global Response Normalization (GRN), respectively, to help models expand and integrate image dimensions and extract important image channels. This paper proposes a lightweight channel attention mechanism called Hybrid Channel Attention (HCA). This attention mechanism calculates weights for different channels in the image and applies weighting to image channels to preserve important ones during channel integration. In this paper, the HCA attention mechanism mainly consists of Nam and ECA modules. As shown in **Figure 5**, the input image is fed into both modules to calculate channel weights, and the resulting two channel weights are applied to the input image channels.

The Nam module uses input normalization to obtain weights for different dimensions in the image (Liu et al., 2021). This paper uses GroupNorm for calculation, as shown in Equation 2. GroupNorm is a method that groups input data based on channel dimensions and then normalize within each group. In the Nam module, mean and variance are calculated for each group and normalized to obtain $W_{\gamma }$ .


(2) $$GN(x)=\gamma \frac{x-\mu_{x}}{\sqrt{\sigma_{x}^{2}+\epsilon }}+\beta$$

(3) $$W_{\gamma }=\frac{\gamma_{i}}{\sum_{j=0}\gamma_{j}}$$

(4) $$W_{nam}=sigmoid\left(W_{g}\left(GN\left(x\right)\right)\right)$$

where $\mu_{x}$ and $\sigma_{x}$ are the mean and variance in each specified grouping, respectively, and the *γ* and *β* are trainable affine transformations. The parameters, $W_{\gamma }$ , are composed of the scaling factor *γ* of each channel and are calculated according to Equation 3. $W_{nam}$ is the channel attention weight obtained from the Nam module, as shown in Equation 4.

The ECA module first applies global average pooling to the input image, then processes the image through a one-dimensional convolution with an adaptively adjustable kernel to generate channel weights (Wang et al., 2020). In the formula, the size of the convolution kernel *k* is determined by the mapping of the channel dimension. The calculation formula is as follows.


(5) $$W_{eca}=sigmoid\left({C1D}_{k}\left(x\right)\right)$$

(6) $$k=y(C)={\left|\frac{\log_{2}(C)}{d}+\frac{b}{d}\right|}_{odd}$$

In Equation 5, *C1D* represents the 1-dimensional convolution processing; in Equation 6, *C* is the given channel dimension, *k* is the adaptive convolution kernel size, *δ*, and b are set to 2 and 1, respectively, and ${\left|t\right|}_{odd}$ represents the odd number closest to *t*.

After obtaining the channel attention weights of the Nam module and the ECA module, the channel attention weights *W* are obtained by combining them, and the calculation process is shown in Equation 7. Finally, the image is weighted using the resulting weight *W* input.


(7) $$W=sigmoid\left(W_{\gamma }\left(GN\left(x\right)\right)\right)\times sigmoid\left({C1D}_{k}\left(x\right)\right)$$

#### 2.3.3 Coordinate-space attention

**Figure 6:**
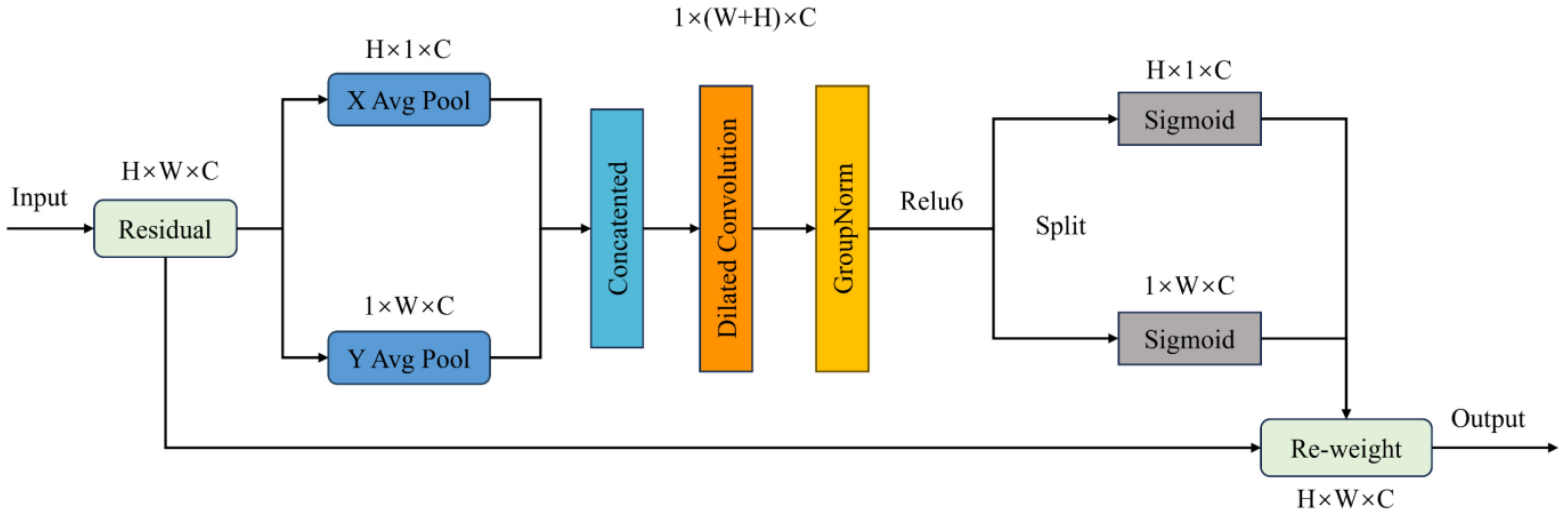
Coordinate space attention mechanism.

In this paper, Coordinate Space Attention (CSA) is a spatial attention mechanism that mainly utilizes spatial position information to obtain weights for different regions in the image. As shown in **Figure 6**, the CSA attention mechanism first applies average pooling to the input image to generate horizontal and vertical feature vectors. Then, the obtained feature vectors are concatenated to form a feature map of the input image. Subsequently, dilated convolution, GroupNorm, and ReLU6 are used to enrich the feature map’s expression and determine whether regions of interest exist in both directions.

The CSA module uses average pooling to obtain horizontal and vertical spatial information of the image, using two spatial pooling kernels (H,1) and (1,W) to encode each channel of the input x along the horizontal and vertical coordinate directions. The height h obtained in channel c can be represented as Equation 8.


(8) $$z_{c}^{h}\left(h\right)=\frac{1}{W}\sum_{0\leq i<W}x_{c}\left(h,i\right)$$

Similarly, the width *w* obtained in the c-channel can be expressed as Equation 9.


(9) $$z_{c}^{w}\left(w\right)=\frac{1}{H}\sum_{0\leq i<h}x_{c}\left(h,w\right)$$

where $z_{c}$ represents the encoded results of h in the horizontal direction w and vertical direction of the c-channel using average pooling, and $x_{c}$ represents the eigenvalues of the c-channel in the feature map at the positions of height h and width w .

Through the calculation Formula 9, the eigenvalues along the abscissa and the ordinate can be obtained. Then, it was re-stitched to obtain a new feature image, and the feature map was processed by using a dilated convolution with a convolution kernel of 3×3. By using dilated convolutions with an expansion rate of 2, it is possible to increase the receptive field without increasing the computational cost of the model. The calculation is shown in Equation 10.


(10) $$f=\delta (F_{3\times 3}^{d}\left(\left[z^{h}{,z}^{w}\right]\right))$$

where [· , ·] Represents a concatenated operation along a spatial dimension. Where $F_{3\times 3}$ is the convolutional transformation function, d is the expansion rate, δ represents the nonlinear activation function, and $f\in R^{C\times \left(H+W\right)}$ is the intermediate feature map that encodes spatial information in the horizontal and vertical directions. Then, the feature map is divided into two independent tensors along the spatial dimension to obtain $f^{h}\in R^{C\times H}$ and $f^{w}\in R^{C\times H}$ . In addition, the attention weights of the tensors $f^{h}$ and $f^{w}$ were obtained by using the sigmoid mapping, respectively, and the calculation formula is shown in Equations 11 and 12.


(11) $$g^{h}=sigmoid\left(F_{h}\left(f^{h}\right)\right)$$

(12) $$g^{w}=sigmoid\left(F_{w}\left(f^{w}\right)\right)$$

Finally, the weights $g^{h}$ and $g^{w}$ are used to weight the input image, and the final result is shown in Equation 13.


(13) $$y\left(i,j\right){=x}_{c}\left(i,j\right)\times g_{c}^{h}\left(i,j\right)\times g_{c}^{w}\left(i,j\right)$$

where y is the final output of CSA, inspired by Coordinate attention, CSA obtains eigenvalues from the horizontal and vertical aspects of the image and processes them. It can help the model to locate the disease location of the leaf in detail. In addition, compared with Coordinate attention, CSA has a smaller number of parameters.

### 2.4 Robust cross-entropy loss

In CNN model training, dataset samples play a crucial role in the training of network model recognition capabilities. However, due to the constraints of environment, equipment, and other factors, the collected plant disease images may contain some data that are difficult to classify, outlier data, and mislabeled data, which will seriously affect the training effect of the model. The Cross-Entropy loss function is a loss function commonly used in classification problems to measure the difference between the model input and the actual label, and for classification problems of M categories, it can be defined as Equation 14.


(14) $$CE\left(p,y\right)=-\sum_{c=1}^{M}y_{\left(o,c\right)}log\left(p_{o,c}\right)$$

where M is the total number of categories, C is the index of the category or class, O is the index of a particular sample in the dataset in the calculation, and when the loss is calculated for a particular sample, c iterates through all possible categories, from 1 to M . $y_{o,c}$ represents the true label of category C in sample O , while the model predicts the probability of category C for sample O when $p_{o,c}$ . If only one category is considered per sample, the $y_{\left(o,c\right)}$ can be treated as 1, and the O index is omitted, the CE loss function can be defined as Equation 15.


(15) $$CE\left(y\right)=-\sum_{c=1}^{M}log\left(p_{c}\right)$$

**Figure 7:**
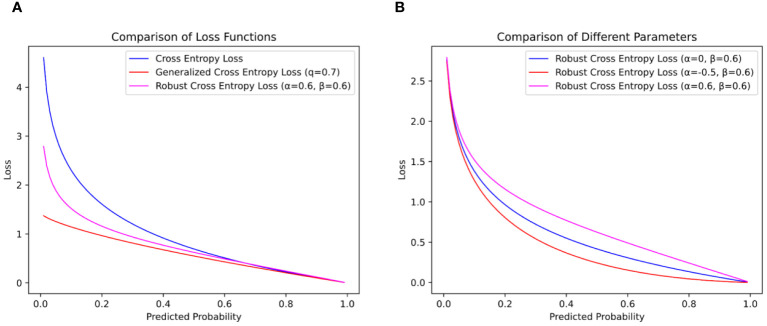
Comparison of loss function curves. **(A)** is the loss function curve of α and β 0.6 and **(B)** is the influence of different α values on the loss function curve.

As shown in **Figure 7**, when the CE loss function is trained, when the accuracy is low, the loss function will give a large loss value to help the model quickly adapt to the image data. This can effectively promote the training of the network model for the dataset with correct labeling, but it will cause the network model to learn wrong data when facing the dataset with noise labels, which will not only lead to the decline of the model’s recognition ability but also make the model over-adapt to the wrong labels, thereby reducing the generalization ability of the model.

To solve this problem, (Zhang and Sabuncu, 2018) proposed Generalized Cross-Entropy Loss (GCE). By introducing the parameter q , the GCE loss function can reduce the penalty by reducing the loss value when the noise is wrong, thereby increasing the tolerance of the model. When q is close to 0, the contribution of the noise label to the total loss is also limited to a small range, reducing the impact on model training. The formula is shown in Equation 16.


(16) $$GCE\left(p,y\right)=\frac{1-p_{y}^{q}}{q}$$

Although the GCE loss function can reduce the impact of noise labels on the recognition ability of the model, it gives a small loss value when the accuracy is high, which makes the model unable to be further trained to improve the recognition ability. In this paper, to help the network model be robust to noise labels in training and improve the effect of model training, a weighted formula with adjustable parameters α and β is introduced to optimize the CE loss function, defined as Equation 17.


(17) $$RCE\left(y\right)=\frac{1}{M}\sum_{c=1}^{M}\left(\left(\alpha \times p_{c}+\beta \right)\times \left(-log\left(p_{c}\right)\right)\right)$$

In the formula, $\left(\alpha \cdot p_{c}+\beta \right)$ represents the added weighting formula. In this weighting formula, α and β are two parameters. Specifically, β serves as a scaling factor that directly influences the overall loss magnitude during model training. When β is greater than 1, the RCE loss function imposes larger penalties; when β is less than 1, it imposes smaller penalties, with β > 0 . On the other hand, parameter α can be used to adjust the magnitude of the loss during model training, constrained by α > −β . A larger α assigns larger losses during training, while a smaller α assigns smaller losses, as depicted in **Figure 7B**.

In this paper, the parameters α and β of the RCE loss function are set to 0.6, as shown in **Figure 7A**. When α and β are 0.6, fewer loss values can be given when the model accuracy is low, and no formal distribution of data is learned, so as to reduce the penalty of the loss function on the noise label, help the model focus on the label with higher confidence, and reduce the influence of the noise label on the model training. When the progress of the model is high, a larger loss value is given, and on the basis of extracting abstract and useful information to a certain extent, the model pays more attention to the samples that are difficult to classify correctly and improves the recognition ability of the model.

### 2.5 Tomato disease identification model process

**Figure 8:**
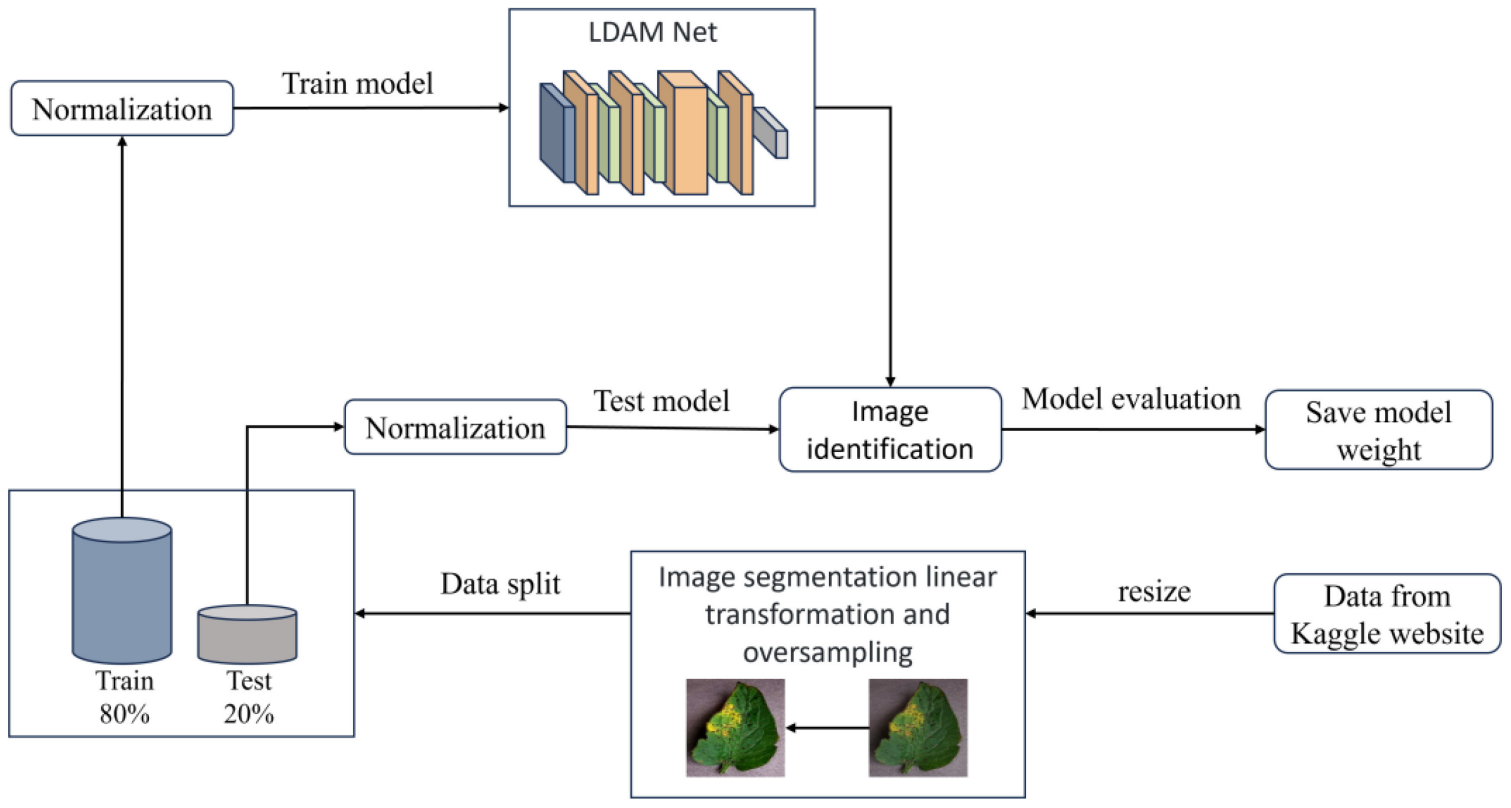
Overall flowchart of LDAMNet model training.

The overall training flow of the LDAMNet model is illustrated in **Figure 8**. In this study, a CNN-based deep learning method is used to construct the recognition model, which heavily relies on the training data. To address issues such as blurred disease features and insufficient image samples in the dataset, this paper first resizes the images and applies piecewise linear transformation to enhance image detail features, as shown in **Figure 8**. After processing the images in the dataset, they are divided into training and test sets at an 8:2 ratio. Then, to improve the model’s generalization ability, normalization is applied to the images in both training and test sets to standardize pixel values across different channels. Finally, to avoid issues caused by interrupted training, this paper saves model parameter files at each training epoch to facilitate continued training. Additionally, to achieve effective disease recognition, the weights of the best-fitting model are saved during training based on set evaluation parameters for subsequent use.

## 3 Experimental results and discussion

### 3.1 Experimental design

The computer used in this paper uses the Windows 11 operating system, uses a 12th Gen Intel(R) Core(TM) i7-12700 (2.10 GHz) processor, and uses a GPU for model training and testing, and the GPU is NVIDIA GeForce RTX 3060(12G). The software environment uses Python 3.9.13, PyTorch 1.13.1, and Cuda 11.6 frameworks.

The experiment is divided into five parts. Namely, the comparative test between different network models proposed in this paper, the comparative test with the inverted bottleneck block, the comparative test using different loss functions, the ablation experiment, and the comparative test on different datasets.

In the training of the neural network model, the Adam stochastic gradient descent method was used to optimize the network model. The calculation of this algorithm is relatively simple, and it has strong adaptability to the gradient. The learning rate is set to 0.0001, the number of iterations is set to num_epochs = 100, and the number of images per batch batch_size = 16. In addition, the AutoAugment method is used to process the training set images, which can enhance the network training effect.

### 3.2 Evaluation indicators

In order to effectively evaluate the trained neural network model, precision, recall, accuracy, and F1 score were used to measure the performance of the neural network model in the identification of tomato leaf diseases. These parameters are calculated as shown in Equations 18–21.


(18) $$Precision=\frac{TP}{TP+FP}$$

(19) $$Recall=\frac{TP}{TP+FN}$$

(20) $$Accuray=\frac{TP+TN}{TP+FN+FP+TN}$$

(21) $$F1=\frac{2TP}{2TP+FP+FN}$$

In the formula, TP (True Positive) is the true example, which indicates the number of positive samples predicted by the model; TN (True Negative) is the true negative example, which indicates the number of negative samples predicted by the model, FP (False Positive) is a false positive example, which indicates the number of negative samples predicted by the model, and FN(False Negative) is a false negative example, which indicates the number of positive samples predicted by the model to be negative.

In this study, precision represents the proportion of samples that are correctly judged to be positive by the network model. Recall measures the proportion of positive class samples correctly identified by the network model in actual positive class samples. Precision represents the ratio of the total number of samples correctly classified by the network model to the total number of samples. The F1 value is a harmonic average of precision and recall, taking into account precision and recall, and is balanced between precision and recall.

In addition, two parameters, Flops (floating-point arithmetic) and Params (number of parameters), are introduced to evaluate the size of the network model. The larger the Flops, the more computational resources the network model needs for training and inference, and this parameter usually represents the Flops computation in a single forward propagation. Params represent the number of parameters in the model, including all weights and biases that need to be learned, and larger Params mean that the larger the network model, the more storage space is needed to hold the model weights.

### 3.3 Experiments of different network models

To test the performance of the LDAMNet network model, this study compares it with ConvNeXtV2 (Woo et al., 2023), Inception_Next (Yu et al., 2023), DenseNet121 (Huang et al., 2017), ResNet18 (He et al., 2016), GhostNet (Han et al., 2020), EfficientNet (Tan and Le, 2019), EfficientFormer (Li et al., 2023), MobileNet (Sandler et al., 2018), MobileVitV2 (Mehta and Rastegari, 2022), Swin Transformer V2 (Liu et al., 2022), Deit3 (Touvron et al., 2022). The models participating in the experiment were evaluated using six parameters: precision, recall, accuracy, F1 score, Flops, and Params. In the comparative experiment, to effectively detect the recognition capabilities of LDAMNet and different network models, these 11 network models were divided into three categories: large-scale CNN models, lightweight CNN models, and Vit models. The large-scale CNN models include ConvNeXt, Inception_Next, DenseNet, and ResNet; the lightweight CNN models include GhostNet, EfficientNet, and MobileNet; the Vit models include EfficientFormer, MobileVitV2, Swin Transformer V2, and Deit3. The accuracy curve comparison of these three types of models with LDAMNet is shown in **Supplementary Figure S1**. The network models used in this experiment are all from the Timm library, and the experimental results are shown in **Table 3**.

**Table 3:** Comparison table of evaluation parameters obtained from training of different network models.

Model	Accuracy	Precision	Recall	F1 score	Flops(G)	Params(M)
ConvNeXt V2_T	94.87	95.02	94.83	94.92	4.45	27.79
Inception_Next_T	97.15	97.30	97.16	97.23	4.2	28.04
DenseNet121	98.64	98.69	98.67	98.68	2.83	7.89
ResNet18	98.05	98.04	97.94	97.99	1.82	11.69
GhostNet V2	94.30	94.53	94.40	94.46	0.42	11.10
EfficientNet	92.08	92.19	92.11	92.15	0.38	5.24
EfficientFormerV2	96.49	96.57	96.50	96.48	1.23	12.63
MobileNetV2	94.19	94.33	94.23	94.28	0.3	3.47
MobileVitV2	95.15	95.25	95.16	95.14	1.41	4.87
Swin TransformerV2	98.22	98.28	98.22	98.22	4.51	28.33
Deit3	97.57	97.60	97.51	97.51	4.24	21.97
LDAMNet(Proposed model)	**98.71**	**98.73**	**98.69**	**98.71**	**0.142**	**0.910**

The bold values represent the best data in the experiment, such as the best average Accuracy, the best average Precision, the best Recall, the best F1 score, the minimum Flops requirement, and the minimum Params requirement.

As shown in **Table 3**, in the comparative experiment, the average values of accuracy, precision, recall, and F1 score of the LDAMNet model are the highest among the eight network models, which are 98.71, 98.73, 98.69, and 98.71, respectively. In addition, the Flops and Params parameters of the LDAMNet model are 0.142 and 0.91, respectively, which are the smallest among the 12 models, indicating that this network model can achieve lightweight and high-precision recognition of tomato diseases.

Furthermore, the experimental results show that on the processed tomato dataset, the recognition ability of the LDAMNet model is higher than that of the other 11 network models. The recognition ability of the LDAMNet model is not significantly different from the large models in this paper; according to the evaluation parameters obtained in **Table 3**, the average accuracy of LDAMNet, DenseNet121, Swin Transformer, and ResNet18 can all reach more than 98%. However, the floating-point operations and parameter counts required by the LDAMNet network model are much smaller than those of the other three types of models, which proves that the LDAMNet model, as a lightweight network model, can achieve the recognition ability of large-scale CNN models or mainstream Vit models, or even slightly better.

In this experiment, the LDAMNet model was compared with three mainstream lightweight models: GhostNet, EfficientNet, and MobileNet. As shown in **Supplementary Figure S1B**, the recognition ability of the LDAMNet model is better than that of these three mainstream lightweight models, with the gap between the EfficientNet model and the LDAMNet model being the largest, with the four evaluation parameters being about 6.63%, 6.54%, 6.58%, and 6.56% higher, respectively.

**Figure 9:**
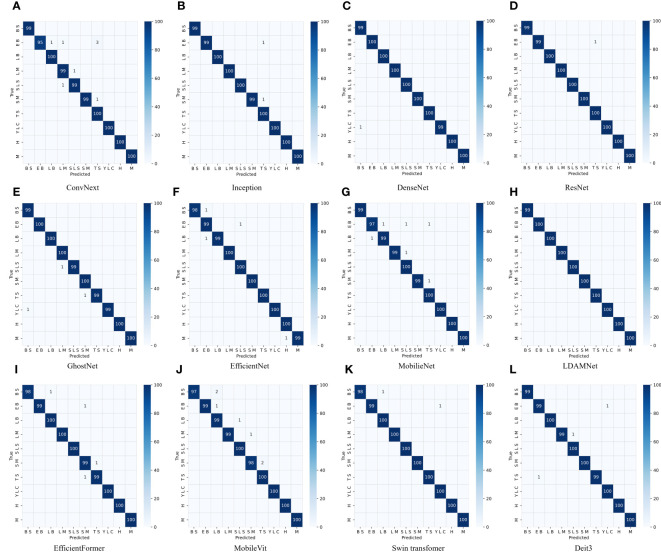
Confusion matrix testing for different CNN models. Models from **(A)** to **(L)** are the models for comparative experiments in **Table 4**, respectively.

Finally, to test the classification effects of the 12 models, a confusion matrix was used to test the models. As shown in **Figure 9**, BS, EB, H, LB, LM, SLS, SM, TS, M, and YLC represent ten types of leaves in the tomato image dataset used in this paper. The test dataset was obtained from the dataset in proportion, including 999 images in 10 categories. In all confusion matrix tests, only the LDAMNet proposed in this paper achieved complete recognition of the test dataset. The other 11 network models all produced a certain number of misjudgments. Among them, ConvNext, MobileNet, and MobileVit had the most misjudgments, with 8, 6, and 6, respectively. EB was the main category of incorrect recognition by the models. The main reason is the uncertain regional distribution of tomato leaf data and the similar characteristics of different types of leaf diseases. For example, both EB and BS diseases produce black or brown spots in appearance, with only slight differences in the shape and color of the spots, leading to some network models being unable to effectively extract features, causing misjudgment.

### 3.4 Experimental of inverted bottlenecks

In subsection 2.2.1, this paper proposes an improved inverted bottleneck block DAC block by adding a channel attention mechanism and a spatial attention mechanism, respectively. In order to verify the improvement effect, the improved inverted bottleneck block (CIB block) in ConvNeXt V2 and the inverted bottleneck block (IB block) used in MobileNet V2 were used for experimental comparison in this experiment, and the three-block structures are shown in **Figure 4**. **Table 4** shows the parameters of the LDAMNet model trained with three blocks, among which the model with DAC block has the highest evaluation parameters, which are 3.34, 3.33, 1.09 and 1.09 larger than the lowest CIB block, respectively. In addition, among the three types of block, most of the parameters required for training are made with the IB block, followed by the CIB block, and finally, the DAC block. The results show that compared with the IB block and CIB block, the DAC block can achieve higher recognition ability with fewer computing resources.

**Table 4:** Parameters were evaluated using the IB, CIB, and DAC block.

Methods	Accuracy	Precision	Recall	F1 score	Flops(G)	Params(M)
IB block	97.57	97.64	97.60	97.62	0.288	1.836
CIB block	95.37	95.49	95.36	95.42	0.189	1.176
DAC block	**98.71**	**98.73**	**98.69**	**98.71**	**0.142**	**0.910**

The bold values represent the best data in the experiment, such as the best average Accuracy, the best average Precision, the best Recall, the best F1 score, the minimum Flops requirement, and the minimum Params requirement.

CIB block, as an improved method of IB block, although the parameters of IB block are effectively reduced by reducing the normalization method and placing the convolutional layer in front of the whole channel, it cannot effectively extract image features in the face of tomato disease image dataset due to its use of 7×7 convolution kernel. The reason is that some diseases in the tomato disease image show small local regions, and although the 7×7 convolutional kernel can obtain the association of regions in space through the receptive field method, it will also lead to inaccurate information acquisition in local regions, which leads to the inferior recognition ability of CIB block in this dataset. However, the IB block and DAC block using the 3×3 convolutional kernel can fully extract the local features of the image so that the recognition ability of the two blocks is similar.

**Figure 10:**
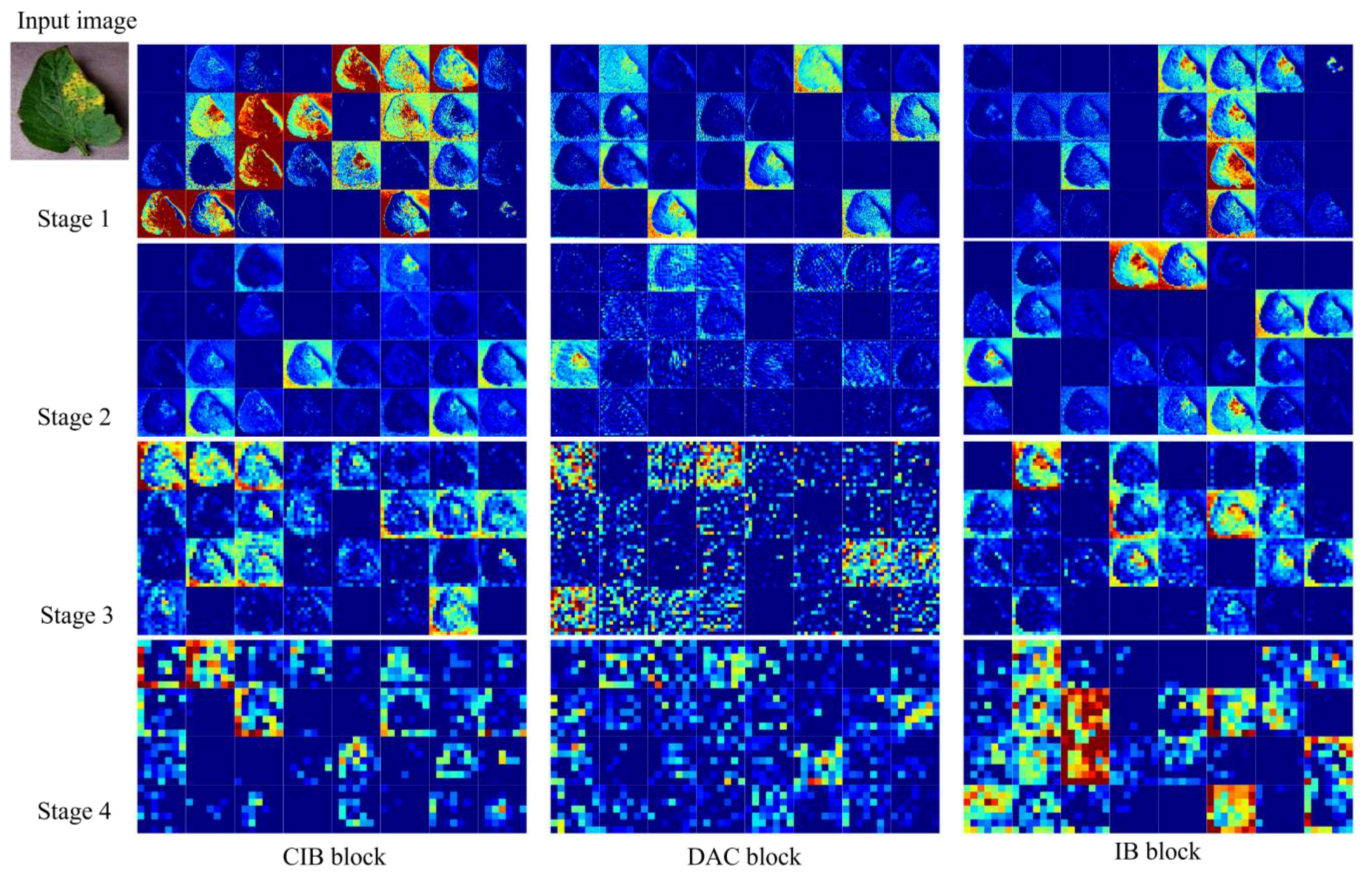
Characteristic diagram of the LDAMNet network at different stages using three blocks.

**Figure 10** shows the feature map of the LDAMNet model using different blocks in four stages. In the first three stages, the CIB block and IB block can better preserve the image outline than the DAC block. However, in the fourth stage, the output feature map contains fewer abstract features than the DAC block, and even some feature maps do not contain image features. As a result, the classifier of the LDAMNet model using CIB block and IB block cannot discriminate the input graph with missing features, which affects the recognition ability of the LDAMNet model.

### 3.5 Experiment of normalization methods

In this paper, in order to reduce the influence of different batches in model training, the GN normalization method is used instead of the BN normalization method commonly used in convolutional neural networks. In addition, in order to verify the optimization effect of the LDAMNet network using the GN method, in this experiment, four normalization methods were used: (Wu and He, 2020), BN (Ioffe and Szegedy, 2015), IN (Ulyanov et al., 2016), and LN (Ba et al., 2016), respectively, in Batch size=8, Batch size=16, and Batch size=32 cases to train the network model.

**Supplementary Figure S2** shows the transformation of the accuracy curve of the LDAMNet network model trained using four normalization methods: GN, BN, LN, and IN. Among the four normalization methods, the accuracy curves using the IN and LN normalization methods fluctuated greatly with different batches. However, the accuracy curve using the GN and BN normalization methods is more stable in the three cases. In addition, as shown in **Table 5**, the GN normalization method can achieve the highest accuracy in the three cases with different batch sizes, while the BN normalization method is slightly lower. Among them, the maximum accuracy difference between GN and BN is 1.05% when Batch size = 16, and the minimum accuracy difference is 0.36% when Batch size = 32.

**Table 5:** Accuracy values of different normalization methods under different batch sizes.

Batch size	Methods	Accuracy	Precision	Recall	F1 score
8	LN	96.69	96.84	96.67	96.75
	IN	96.22	96.30	96.20	96.25
	BN	97.68	97.81	97.65	97.73
	GN	**98.49**	**98.64**	**98.51**	**98.57**
16	LN	96.28	96.48	96.34	96.41
	IN	96.34	96.52	96.35	96.43
	BN	97.66	97.75	97.63	97.69
	GN	**98.71**	**98.73**	**98.69**	**98.71**
32	LN	96.66	96.87	96.63	96.75
	IN	96.03	96.25	96.02	96.13
	BN	97.88	97.94	97.89	97.91
	GN	**98.24**	**98.38**	**98.25**	**98.31**

The bold values represent the best data in the experiment, such as the best average Accuracy, the best average Precision, the best Recall, the best F1 score, the minimum Flops requirement, and the minimum Params requirement.

### 3.6 Ablation experiments

In this section, we conducted ablation experiments, comparison experiments of different attentional mechanisms, and experiments using different loss functions for training. As shown in **Supplementary Figure S3A**, among the three improvements of HCA, CSA, and DAC, the DAC block has the greatest improvement in the recognition performance of the network, followed by the HCA and CSA modules. As shown in **Table 6**, the DAC block, which aggregates CSA and HCA in the lightweight LDAMNet model, can effectively help the network model improve the recognition accuracy of the disease, and its average accuracy can reach 98.71%. The average recognition accuracy of the HCA block and CSA block is 96.21% and 95.89% respectively, which indicates that both of them can effectively improve the recognition ability of the model in LDAMNet, with the attention effect of HCA being slightly better.

**Table 6:** Comparative experiments of different structures and training methods of network models.

Settings	Accuracy	Precision	Recall	F1 score	Flops(G)	Param(M)
Baseline	95.19	95.31	95.18	95.24	**0.1418**	**0.9057**
+HCA	96.21	96.31	96.18	96.24	0.1425	0.9057
+CSA	95.89	95.97	95.88	95.92	0.1419	0.9105
+CA	97.98	98.03	97.92	97.97	0.1422	0.9385
+CBAM	97.34	97.41	97.31	97.36	0.1424	0.9279
+DAC(CE)	98.15	98.27	98.20	98.23	0.1426	0.9105
+DAC(RCE)	**98.71**	**98.73**	**98.69**	**98.71**	0.1426	0.9105

The bold values represent the best data in the experiment, such as the best average Accuracy, the best average Precision, the best Recall, the best F1 score, the minimum Flops requirement, and the minimum Params requirement.

**Figure 11:**
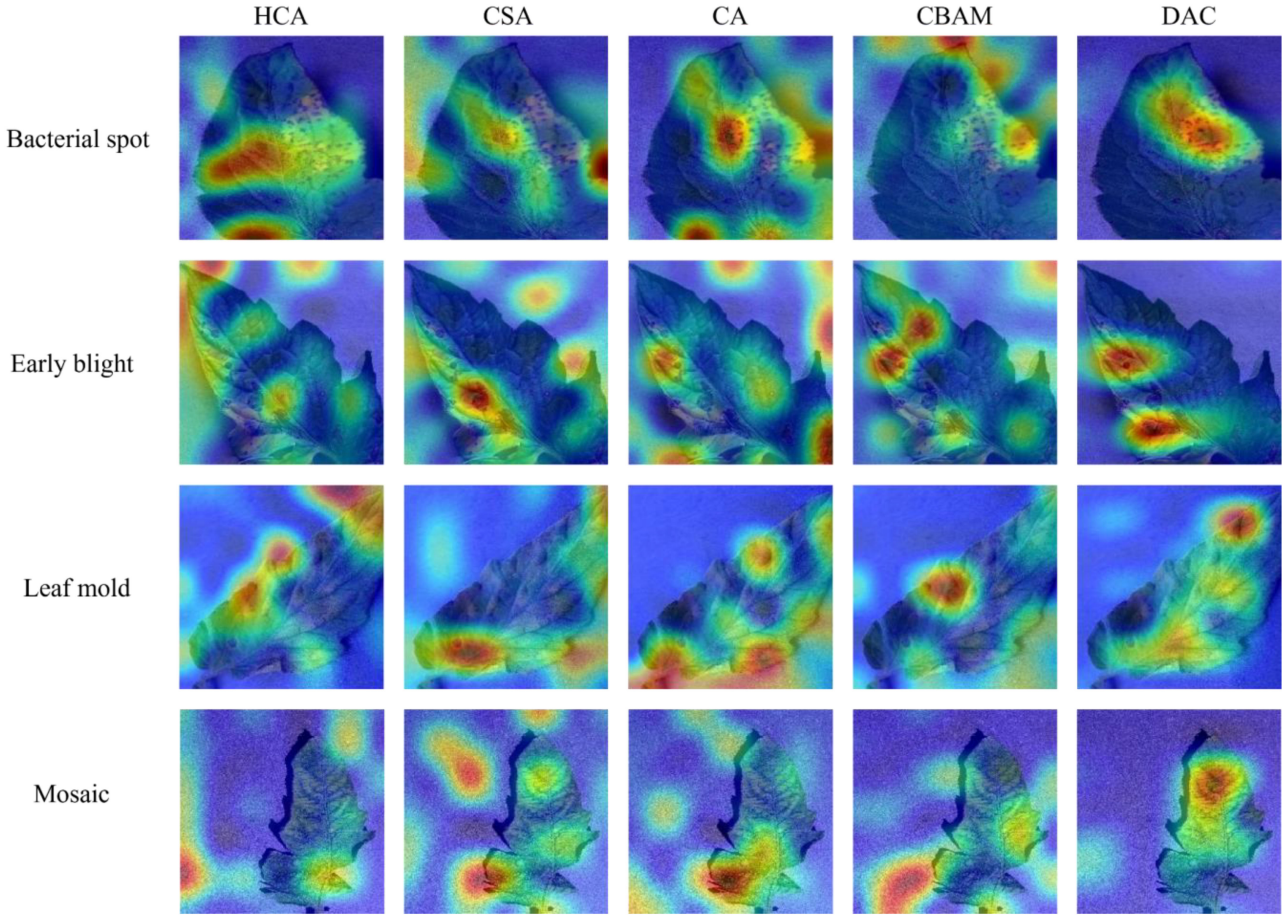
Category activation diagram using different attention mechanisms in the face of four different tomato diseases.

Then, to examine the difference between the DAC block proposed in this paper and mainstream attention mechanisms, CA and CBAM were introduced for comparison experiments. **Supplementary Figure S3B** shows the variation of the experimental accuracy curves, in which the CA and CBAM attention mechanisms have some fluctuations in their accuracy curves during the training cycle, while the DAC accuracy curve is relatively smooth. The test data, as shown in **Table 6**, show that there is no significant difference among the three methods in terms of the amount of computation and the number of parameters required, while the average accuracy of the DAC block method is slightly higher than that of the two attention mechanisms, CA and CBAM. **Figure 11** shows the class activation diagrams of the LDAMNet network model using different attention mechanisms, and the input images are the four leaf disease images in **Figure 1**. From the figure, it is clearly observed that the DAC block effectively captures the leaf disease regions at different locations, whereas HCA, CSA, CA, and CBAM attention mechanisms do not capture the regions as accurately as the DAC block.

Finally, in this section, to validate the RCE loss function proposed in this paper, the mainstream CE loss function is used for comparison. **Supplementary Figure S3C** shows the comparison of the accuracy curves of LDAMNet using the RCE loss function and the CE loss function, respectively. The LDAMNet model applying the RCE loss function does not have the effect of too small loss values in the pre-training period, which leads to slower convergence, and its accuracy curve is more stable in the late training period.

### 3.7 Cross-dataset experiments

Through the above experiments, it can be proved that the model proposed in this paper has a strong recognition ability in the tomato dataset. However, it is unknown whether the model can have the same advantages in the face of different leaf disease datasets. Therefore, in this experiment, in order to test the recognition ability of the LDAMNet network model in the face of different leaf disease images, the model was trained and tested using Rice Leaf Disease Images with complex backgrounds, and the samples of the rice dataset are shown in **Supplementary Figure S4**, including Bacterialbight, Blast, Brownsport, Tungro has a total of 5932 images (https://www.kaggle.com/datasets/nirmalsankalana/rice-leaf-disease-image) in four categories.

**Figure 12:**
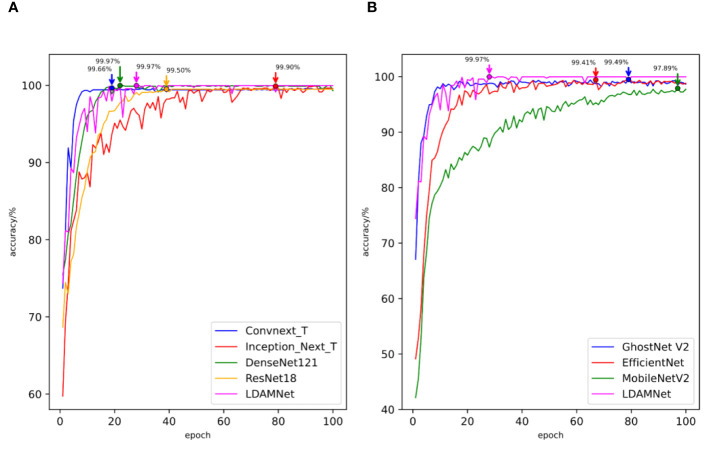
The accuracy curves of different models trained using rice datasets. **(A)** comparison of the accuracy curves of large-scale models **(B)** and comparison of the accuracy curves of lightweight models.

In order to detect the gap between the recognition ability of the LDAMNet model in this dataset and the current mainstream models, the seven models used in Part 3.3 were used in this experiment. In the experiment, set the Batch Size to 16, the training round epoch to 100, and the learning rate to 0.00001. **Table 7** lists the number of datasets. In addition, ConvNeXt, Inception, DenseNet, ResNet, GhostNet, EfficientNet, and MobileNet were trained using the CE loss function, and LDAMNet was trained using the RCE loss function, and the evaluation parameters obtained from the test are shown in **Table 8**, and the change of the accuracy curve is shown in **Figure 12**.

**Table 7:** Number of samples from the training and test sets of rice image datasets without data augmentation.

Categories	Bacterial blight	Blast	Brownsport	Tungro
Train	1268	1152	1280	1046
Test	316	288	320	262

**Table 8:** Comparison of evaluation parameters obtained by the model trained using the rice dataset.

	Model	Accuracy	Precision	Recall	F1 score	Flops(G)	Params(M)
1	Connect V2_T	98.44	**98.71**	98.46	98.58	4.45	27.79
2	Inception_Next_T	96.10	96.35	96.20	96.27	4.2	28.04
3	DenseNet121	98.52	98.51	98.51	98.51	2.83	7.89
4	ResNet18	97.16	97.28	97.19	97.24	1.82	11.69
5	GhostNet V2	97.95	97.95	97.95	97.95	0.42	11.10
6	EfficientNet	95.56	95.47	95.53	95.50	0.38	5.24
7	MobileNetV2	90.44	90.40	90.59	90.49	0.3	3.47
8	LDAMNet(Proposed model)	**98.56**	98.70	**98.58**	**98.63**	**0.142**	**0.910**

The bold values represent the best data in the experiment, such as the best average Accuracy, the best average Precision, the best Recall, the best F1 score, the minimum Flops requirement, and the minimum Params requirement.

The measured data are shown in **Table 8**, and the highest scores of the Accuracy, Recall, and F1 scores of the model are 98.56, 98.58, and 98.65, respectively, while the Precision parameter of the ConvNeXt model achieves the highest value of 98.71, which is slightly higher than the 98.70 of the LDAMNet model. The measured data show that LDAMNet can still maintain the same recognition ability as the existing mainstream large-scale models after replacing it with the rice dataset and can also maintain certain advantages compared with the lightweight model.

**Figure 12** shows the accuracy curves of the different models in the experiment. As shown in the figure, the recognition accuracy convergence speed of the proposed model in the early stage of training is relatively slow and fluctuates to a certain extent. However, with further training of the model, the recognition accuracy of the LDAMNet model can be stabilized in a high region. The results show that the network model proposed in this paper can still maintain high recognition performance in the face of cross-dataset and has a certain generalization.

## 4 Conclusion

This paper addresses the issues of uneven distribution of disease features in tomato leaf images, significant differences within similar features, and small differences between dissimilar features. A high-precision and lightweight leaf disease recognition method has been designed. First, linear transformation is used to enhance the image, augmenting the detail features of the disease and mitigating the problems of significant differences within similar features and small differences between dissimilar features. Then, DAC block, composed of HCA, CSA, and IBA blocks, is used to build a lightweight network model called LDAMNet. Additionally, the RCE loss function is employed to train the model, increasing its robustness. Comprehensive testing shows that this method can effectively identify tomato leaf diseases, offering certain advantages over mainstream large-scale and lightweight models, with maximum accuracy, precision, recall, and F1 scores reaching 99.88, 99.88, and 99.87, respectively. This confirms that LDAMNet achieves high-precision disease recognition while being a lightweight model.

Moreover, to verify the generalization of this detection method, a rice disease dataset was used for testing. Experimental results indicate that the proposed method still maintains certain advantages and can be used for cross-dataset disease recognition. Although LDAMNet achieves high-precision disease recognition, it still has potential for further exploration. Its average recognition accuracy on the rice disease dataset has not reached the optimum level. Further improvements are needed to address the issue of uneven distribution of disease features in complex backgrounds.

In summary, this paper proposes a method for detecting tomato leaf diseases and establishes a new lightweight convolutional neural network model, LDAMNet. Tests have shown that this model can effectively identify tomato leaf diseases and maintain strong recognition capability even in the complex backgrounds of the rice disease dataset. The proposed method can effectively identify agricultural leaf diseases, providing a feasible approach for early identification and reasonable treatment of agricultural diseases.

## Data availability statement

The raw data supporting the conclusions of this article will be made available by the authors, without undue reservation.

## Author contributions

EZ: Writing – original draft. NZ: Writing – review & editing. FL: Resources, Writing – original draft. CL: Visualization, Writing – original draft.
