# Supplementary material for: A lightweight dual-attention network for tomato leaf disease identification
Source: Front Plant Sci. 2024 Aug 6;15:1420584. doi: 10.3389/fpls.2024.1420584 (PMC11333365; doi:10.3389/fpls.2024.1420584)
Supplement: Supplementary file 1 [file Table_1.docx]

**Table S1**  Comparison table of abbreviations of full names in the text

| Full name | Abbreviation | Full name | Abbreviation | Full name | Abbreviation |
| --- | --- | --- | --- | --- | --- |
| Lightweight Dual Attention Mechanism Network | LDAMNet | ConvNeXt Inverted bottleneck block | CIB block | Healthy | H |
| Dual Attention Convolution Block | DAC block | Inverted bottleneck block | IB block | Late blight | LB |
| Inverted Bottleneck Attention Block | IBA block | Layer Normalization | LN | Leaf mold | LM |
| Hybrid Channel Attention block | HCA | Instance Normalization | IN | Septoria leaf spot | SLS |
| Coordinate Space Attention Block | CSA block | Batch Normalization | BN | Spider mites | SM |
| Robust Cross-Entropy | RCE | Group Normalization | GN | Target spot | TS |
| Cross-Entropy | CE | Bacterial spot | BS | Mosaic | M |
| Generalized Cross-Entropy | GCE | Early blight | EB | Yellow leaf curl | YLC |

**Table S2** Disease characteristics of different tomato image categories

|  | Disease | Disease Characterization |
| --- | --- | --- |
| 1 | Bacterial spot | This is characterized by the presence of small, water-soaked spots on leaves, stems, and fruits, which subsequently darken to brown or black with irregular margins. Under moist conditions, a yellowish ooze may develop at the center of these spots. |
| 2 | Early blight | This pathogen affects leaves and stems, initially manifesting as small black spots that expand into circular or irregular patches with a greyish-brown center and dark edges, resembling a “target”. |
| 3 | Healthy | Healthy tomato plants exhibit deep green leaves devoid of spots or damage, robust stems, and fully colored fruits without cracks or rot. |
| 4 | Late blight | Manifests as large, wet, dark brown to black lesions on leaves with white fungal growth along the margins. |
| 5 | Leaf mold | Manifests as yellow or pale green spots on the upper surface of leaves and a purple to grey mold on the underside. This condition is particularly severe in high-humidity environments, resulting in leaf yellowing and subsequent shedding. |
| 6 | Septoria leaf spot | Small, circular spots with a greyish-white center and dark brown or purple edges, possibly accompanied by black dots (spore-bearing bodies), manifest on leaves. This disease rapidly spreads in moist conditions. |
| 7 | Spider mites | Its characteristic manifestations include yellowish small spots, webbing encasement, and leaf curvature and deformation. Following the onset of the disease, the spots on the leaves gradually spread and turn yellow, ultimately desiccating and shedding. |
| 8 | Target spot | Circular to irregularly shaped dark brown to black spots appear on leaves and fruits. The centers often fall out, forming holes, which lead to early leaf drop. |
| 9 | Mosaic | Leaves display irregular light green to yellow patches, accompanied by stunted growth and deformities. Mosaic is typically caused by a virus and spread by insects. |
| 10 | Yellow leaf curl | Leaves turn yellow and curl inward, exhibiting stunted growth and a significant reduction in fruit quantity and quality. This disease is usually viral and spread by insects. |
