# Supplementary figures and images for: A lightweight dual-attention network for tomato leaf disease identification
Source: Front Plant Sci. 2024 Aug 6;15:1420584. doi: 10.3389/fpls.2024.1420584 (PMC11333365; doi:10.3389/fpls.2024.1420584)

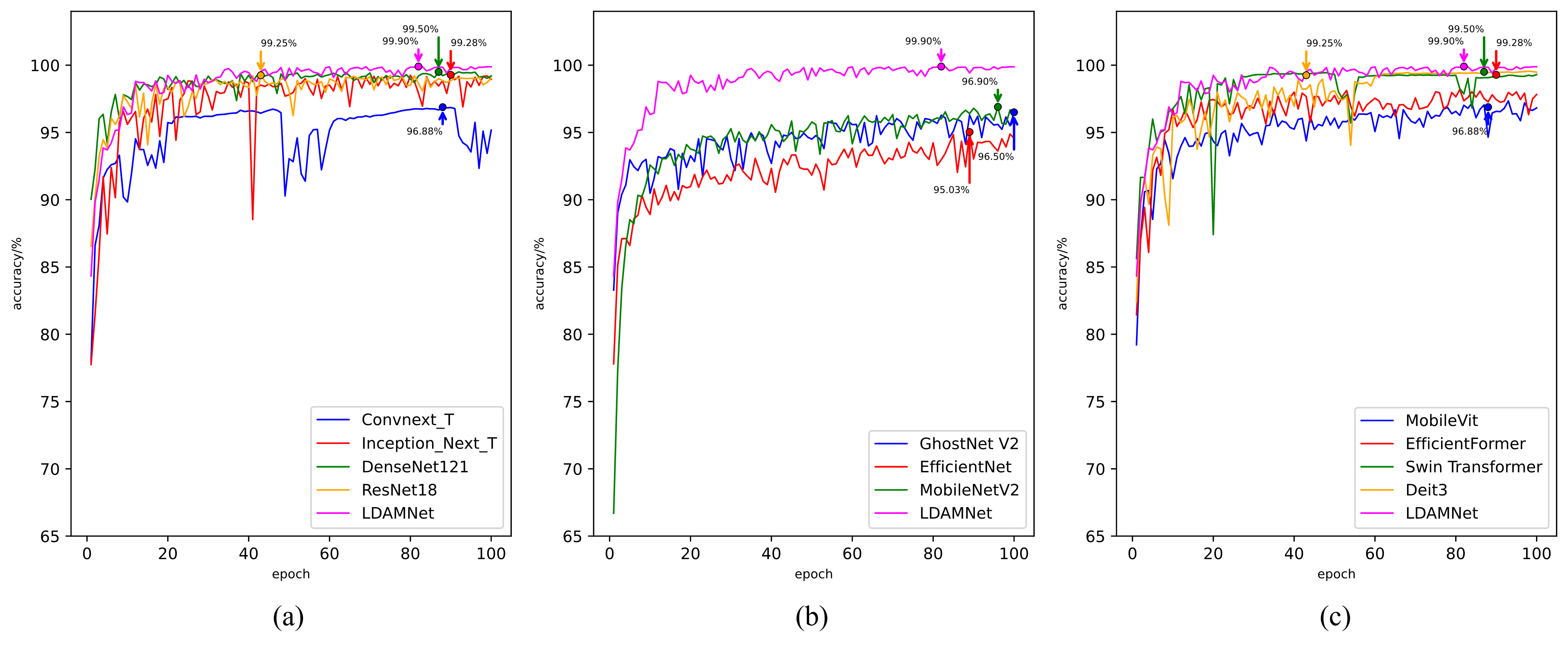

Supplement: Supplementary file 2 [file Image_1.tif]

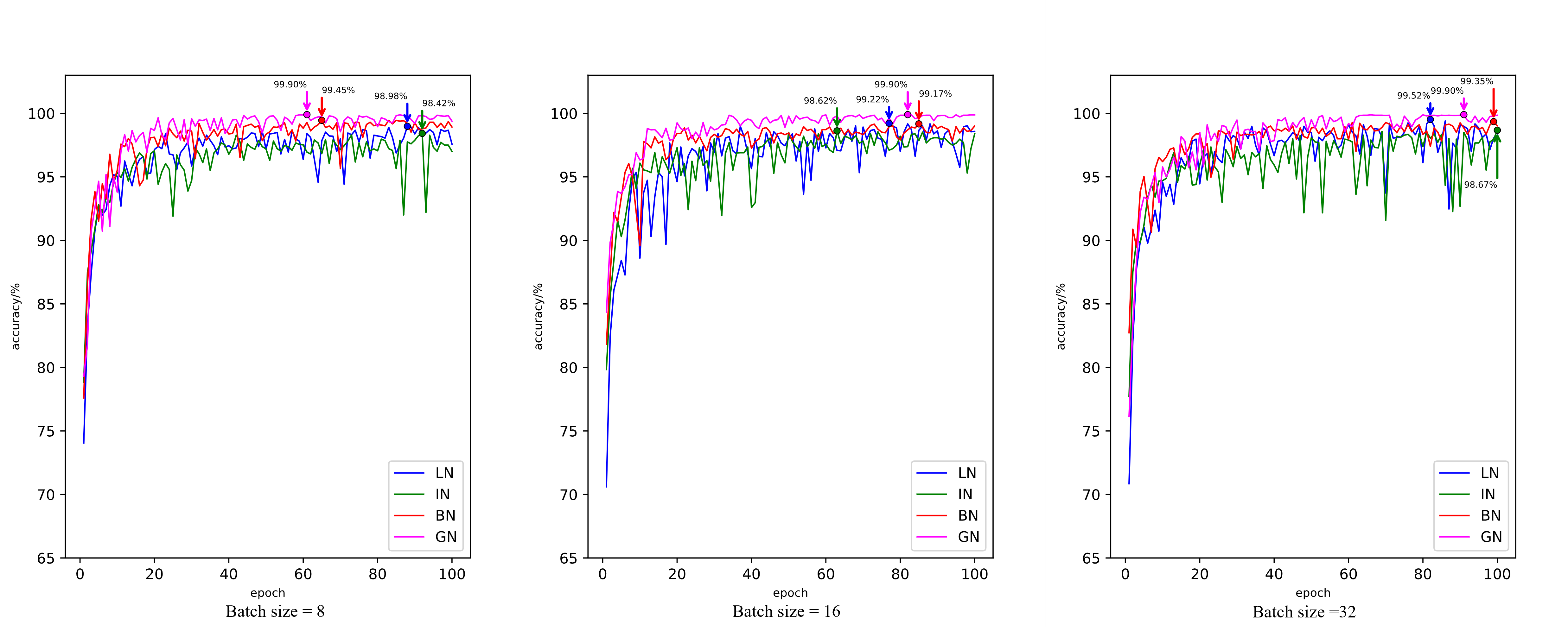

Supplement: Supplementary file 3 [file Image_2.tif]

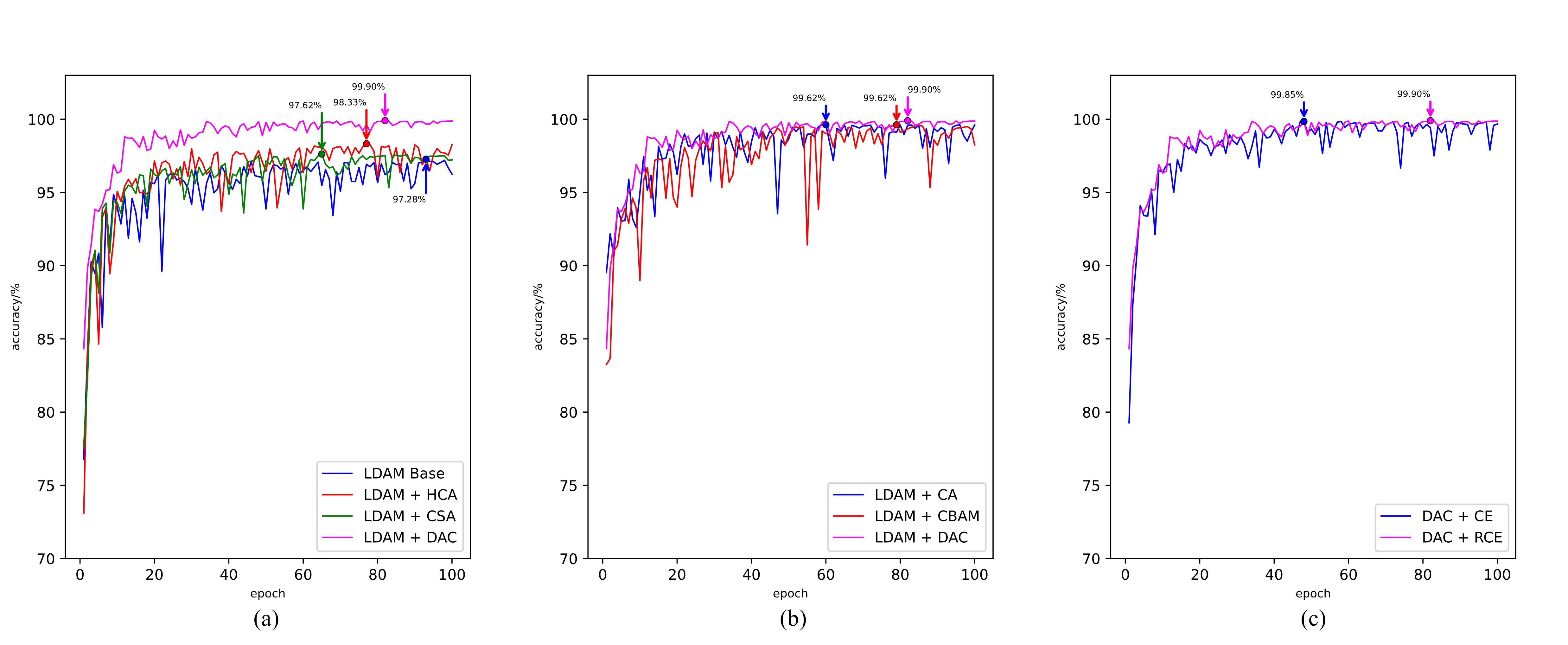

Supplement: Supplementary file 4 [file Image_3.tif]

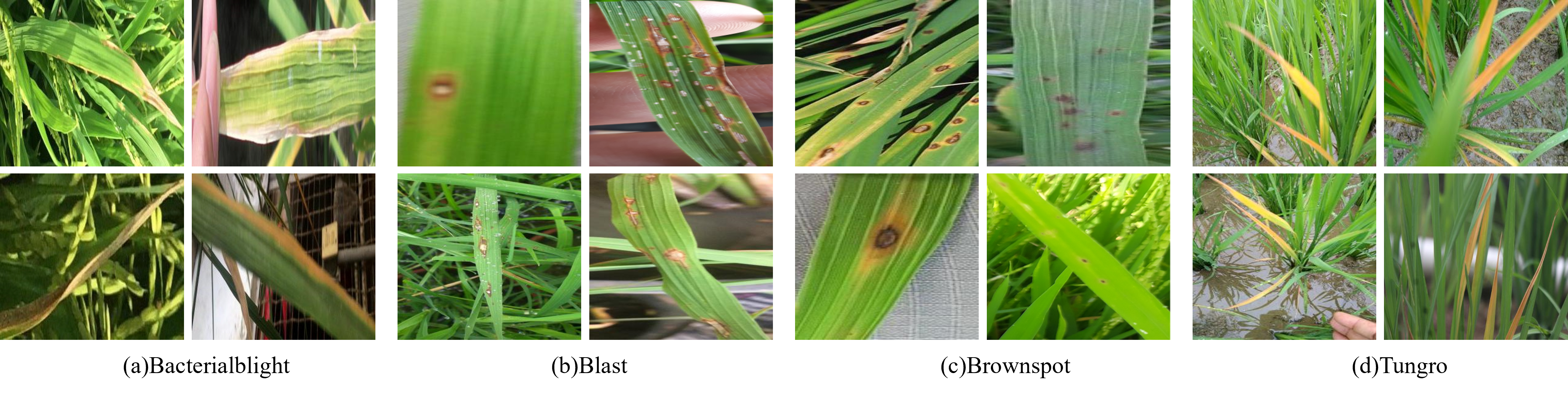

Supplement: Supplementary file 5 [file Image_4.tif]
